# Balance of power: The choice between trial and participant numbers to optimise the detection of phase-dependent effects

**DOI:** 10.1162/imag_a_00345

**Published:** 2024-11-05

**Authors:** Asher Geffen, Nicholas Bland, Martin V. Sale

**Affiliations:** School of Health and Rehabilitation Sciences, The University of Queensland, St Lucia, QLD, Australia; School of Psychology, The University of Queensland, St Lucia, QLD, Australia; Queensland Brain Institute, The University of Queensland, St Lucia, QLD, Australia

**Keywords:** sample size, participants, trials, power, phase

## Abstract

The fields of neuroscience and psychology are currently in the midst of aso-called reproducibility crisis, with growing concerns regarding a history ofweak effect sizes and low statistical power in much of the research published inthese fields over the last few decades. Whilst the traditional approach foraddressing this criticism has been to increase*participant*sample sizes, there are many research contexts in which the number of*trials*per participant may be of equal importance. Thepresent study aimed to compare the relative importance of participants andtrials in the detection of phase-dependent phenomena, which are measured acrossa range of neuroscientific contexts (e.g., neural oscillations, non-invasivebrain stimulation). This was achievable within a simulated environment where onecan manipulate the strength of this phase dependency in two types of outcomevariables: one with normally distributed residuals (idealistic) and onecomparable with motor-evoked potentials (an MEP-like variable). We compared thestatistical power across thousands of experiments with the same number ofsessions per experiment but with different proportions of participants andnumber of sessions per participant (30 participants × 1 session, 15participants × 2 sessions, and 10 participants × 3 sessions), withthe trials being pooled across sessions for each participant. These simulationswere performed for both outcome variables (idealistic and MEP-like) and fourdifferent effect sizes (0.075—“weak,”0.1—“moderate,” 0.125—“strong,”0.15—“very strong”), as well as separate control scenarioswith no true effect. Across all scenarios with (true) discoverable effects, andfor both outcome types, there was a statistical benefit for experimentsmaximising the number of trials rather than the number of participants (i.e., itwas always beneficial to recruit fewer participants but have them complete moretrials). These findings emphasise the importance of obtaining sufficientindividual-level data rather than simply increasing number of participants.

## 1 Introduction

Neuroscience and psychology studies often yield statistical powers well below thetypically desired level of 80%, with one review reporting a range of only8–30% (Button et al., 2013). This hasled to concern regarding the reproducibility of findings reported in these fields,the most damning of which comes from the Open Science Collaboration (Open Science Collaboration, 2015), which involved 100replications of published psychology experiments and found only 36% of thosereplications were successful. Whilst there are many factors that can influencereplicability, in terms of both experimental design and statistical analysis, one ofthe most commonly raised concerns is the small sample sizes that have traditionallybeen employed by many studies in these fields (Turner et al., 2018). Conventional considerations for maximisingstatistical power focus on increasing the*participant*sample size,since recruiting a greater number of participants will invariably aide the detectionof an experimental effect (Minarik et al.,2016;Mitra et al., 2019). In manyresearch contexts, however, there may be value in considering the number of*trials*within an experimental paradigm (Baker et al., 2021;Normand, 2016;Smith & Little,2018;Zoefel et al., 2019).

The relative importance of each of these factors for any given experiment isdependent on the amount of variability that is expected to be present both withinand between participants (Baker et al., 2021;Grice et al., 2017;Rouder & Haaf, 2018;Xu et al., 2018). For example, if a lot of variability isexpected across trials for a given individual (i.e., when the measured variable isdynamic/unstable), there is an obvious value in ensuring a sufficient amount of dataare collected from each individual to ensure that the individual-level measures areaccurate, rather than simply recruiting more individuals. In this scenario, it isimportant to remember that even with a sufficiently large participant sample size,the group-level analyses are unlikely to be meaningful if the measures at theindividual level are inaccurate (Normand,2016). However, if there is little variability expected across trials(i.e., when the measured variable is static/stable), then there is less value incollecting large number of trials for each participant, with resources insteadallocated towards increasing the participant sample size.

There are many branches of neuroscience research that are particularly prone toconsiderable within- and between-subjects variability, one example of which is thefield of non-invasive brain stimulation (NIBS). NIBS methods make promising toolsfor both studying and modulating brain function (Begemann et al., 2020;de Boer et al.,2021;Lewis et al., 2016;Vosskuhl et al., 2018) that have receivedincreasing interest in recent years for their potential uses in both psychiatry(Elyamany et al., 2021;Piccoli et al., 2022;Vicario et al., 2019) and neurorehabilitation (Evancho et al., 2023;Qiet al., 2023;Yang et al., 2024).Unfortunately, however, studies involving NIBS have traditionally employedrelatively small sample sizes (Button et al.,2013;Minarik et al., 2016;Mitra et al., 2019), which when combined withthe aforementioned within- and between-subjects variability can lead to suboptimalanalyses, inconsistent findings across studies, and ultimately criticism regardingthe efficacy of these stimulation techniques (Lafonet al., 2017;Vöröslakos etal., 2018).

Most of this criticism has historically been directed towards low participant samplesizes (Mitra et al., 2019); however, asmentioned earlier, it has recently been suggested that in many cases, the trialsample size may be of equal, if not greater importance (Baker et al., 2021;Normand, 2016;Rouder & Haaf,2018;Smith & Little, 2018;Xu et al., 2018). For example, a recentsimulation study byZoefel et al. (2019)found that the most important of their experimental parameters for detecting phasicmodulation of brain activity was indeed the number of trials per participant andthey, therefore, suggested that future studies should employ experimental designswith a relatively high number of trials. The authors also found that the mostimportant of their neural parameters for detecting phasic effects was thehypothesised effect size. Therefore, optimising the trial sample size would be ofparticular importance for studies investigating subtle (weak) phasic effects.

This brings us to transcranial alternating current stimulation (tACS), which is aform of NIBS that involves the application of a weak alternating electric currentacross the scalp at the same frequency as a particular neural oscillation in orderto influence the underlying oscillatory activity (Bland & Sale, 2019). Converging evidence suggests that tACS caneffectively modulate oscillatory brain activity in a frequency- and phase-dependentmanner by entraining endogenous oscillations to match the frequency and phase of theexogenous stimulation (for review, seeWischnewskiet al. (2023)). However, because tACS only probabilistically influencesthe spike timing of neuronal populations rather than directly causing those neuronsto depolarise (Elyamany et al., 2021;Huang et al., 2017;Opitz et al., 2016;Vosskuhl et al., 2018), this phasic entrainment is often reported to berelatively weak (Gundlach et al., 2016;Neuling et al., 2012;Riecke, Formisano, et al., 2015;Riecke et al., 2018;Riecke, Sack, et al., 2015;Riecke& Zoefel, 2018;Wilsch et al.,2018;Zoefel et al., 2018).

One approach for investigating phasic entrainment by tACS is to collect transcranialmagnetic stimulation (TMS)-induced motor-evoked potentials (MEPs) at differentphases of tACS, referred to as phase-dependent TMS (Fehér et al., 2017,^2022^;Nakazono et al., 2021;Raco et al., 2016;Schaworonkow et al., 2018,^2019^;Schilberg et al., 2018;Zrenner et al., 2018,^2022^). This allows corticospinal excitability to be probed across tACSphase without the interference of tACS artefacts, which are a major contaminant ofneurophysiological recordings from electro-/magnetoencephalography (Kasten & Herrmann, 2019;Noury et al., 2016;Noury& Siegel, 2017). This technical advantage does come at a cost,however, as MEP amplitudes often exhibit considerable within-participant variabilityacross trials (Capaday, 2021;Janssens & Sack, 2021) due to a complexcombination of physiological (Gandevia &Rothwell, 1987;Hashimoto &Rothwell, 1999;Niyazov et al.,2005;Vidaurre et al., 2017;Zalesky et al., 2014) and experimental factors(Grey & van de Ruit, 2017). Thecombination of small effect sizes for tACS and high inter-trial variability for TMS,therefore, means that the MEP sample size needs to be sufficiently large in order todetect any modulation of the MEP amplitudes with respect to tACS phase. Crucially,however, the maximum number of MEPs that can be obtained in a single experimentsession is limited by several practical limitations, such as the session length,charge time between TMS pulses, and the gradual build-up of the TMS device’stemperature (which can eventually cause the device to overheat).

In a recent human study using slow-wave tACS (Geffenet al., 2021), we acquired a total of 240 MEPs within the tACS protocolof each session. Since these MEPs were acquired across 4 different epochs of thetACS protocol (early online, late online, early offline, and late offline), thisgave us only 60 MEPs per epoch, per participant. Despite employing a participantsample size of 30, which is nearly 50% greater than the mean participant sample sizefrom a recent meta-analysis of NIBS studies (~22;Mitra et al., 2019), the trial sample size could be considered low(e.g., the lowest number of trials was 192 in Zoefel et al.’s simulations in2019). This low trial sample size may ultimately limit the interpretation of theseresults since it cannot be confirmed whether the lack of phasic effects was due toinsufficient statistical power. Might these effects have been detectable had weinstead recruited just 10 participants and had them return for three sessions?

One possible solution to this limit in MEP acquisition is to perform multiplesessions for each participant then pooling the trial data across sessions. However,given that most studies have resource and/or time constraints that limit theirexperimental hours, this approach would come at the cost of participant sample size.Although the simulations performed byZoefel et al.(2019)have demonstrated that trial sample size is a crucial experimentalparameter for detecting phasic effects, those simulations kept participant samplesize constant for all experiments. The relationship between participant and trialsample size for detecting phasic effects thus remains to be established and it isunclear whether the gain of statistical power from increasing the trial sample sizewould outweigh the loss of statistical power from decreasing the participant samplesize. Therefore, we chose to perform a simulation study to quantify the statisticalpowers of experiments that have the same number of total sessions per experiment butwith different proportions of participants and number of sessions per participant.In this manner, the theoretical burden to the researcher (in terms of experimentalhours) is matched in each scenario: a greater number of individuals contribute fewerexperimental sessions or fewer individuals contribute multiple sessions (i.e., agreater number of trials for a given participant pooled across sessions).

## 2 Methodology

### 2.1 Simulation scenarios

All simulations were performed in MATLAB (R2020b). For each scenario, we definedthe type of data (“idealistic” or “MEP-like”), meaneffect size (0.075—“weak,”0.1—“moderate,” 0.125—“strong,” or0.15—“very strong”), number of participants (30, 15, or10), sessions per participant (1, 2, or 3 sessions for 30, 15, and 10participants, respectively), trials per session (60), number of experiments(1000), and the relative degree of between- and within-subjects variability(low, medium, or high).

The total number of sessions and the trials per session were chosen based on theparticipant and trial sample sizes used in our recent slow-wave tACS study (seeabove;Geffen et al., 2021). The effectsizes do not reflect a traditional effect size value such as a Cohen’s*d*value, but rather determine either the amplitude of thebase sine function (in the case of the idealistic data) or the variance of anormal distribution around the base sine function (in the case of the MEP-likedata). The mean values for each effect size category were chosen from pilottesting to provide sinusoidal data with a good spread of statistical power: fromweak effects (e.g., 20% power) to strong effects (e.g., 90% power). As anegative control, we performed a separate test with no effect of phase for anyof the sessions.

### 2.2 Determining effect sizes for each session

For each scenario, separate effect sizes were first generated for eachparticipant that ranged around the mean effect size for the chosen effect sizecategory (i.e., 0.075—“weak,”0.1—“moderate,” 0.125—“strong,” or0.15—“very strong”). The range for the participant effectsizes was set to either ±20%, 60%, or 100% around this mean value forlow, medium, and high between-subjects variability, respectively. The effectsizes for each participant were then jittered slightly between their individualsessions by either ±10%, 20%, or 30% around the value from their firstsession for low, medium, and high within-subjects variability, respectively.

### 2.3 Generating sinusoidal data

**Fig. 1.**
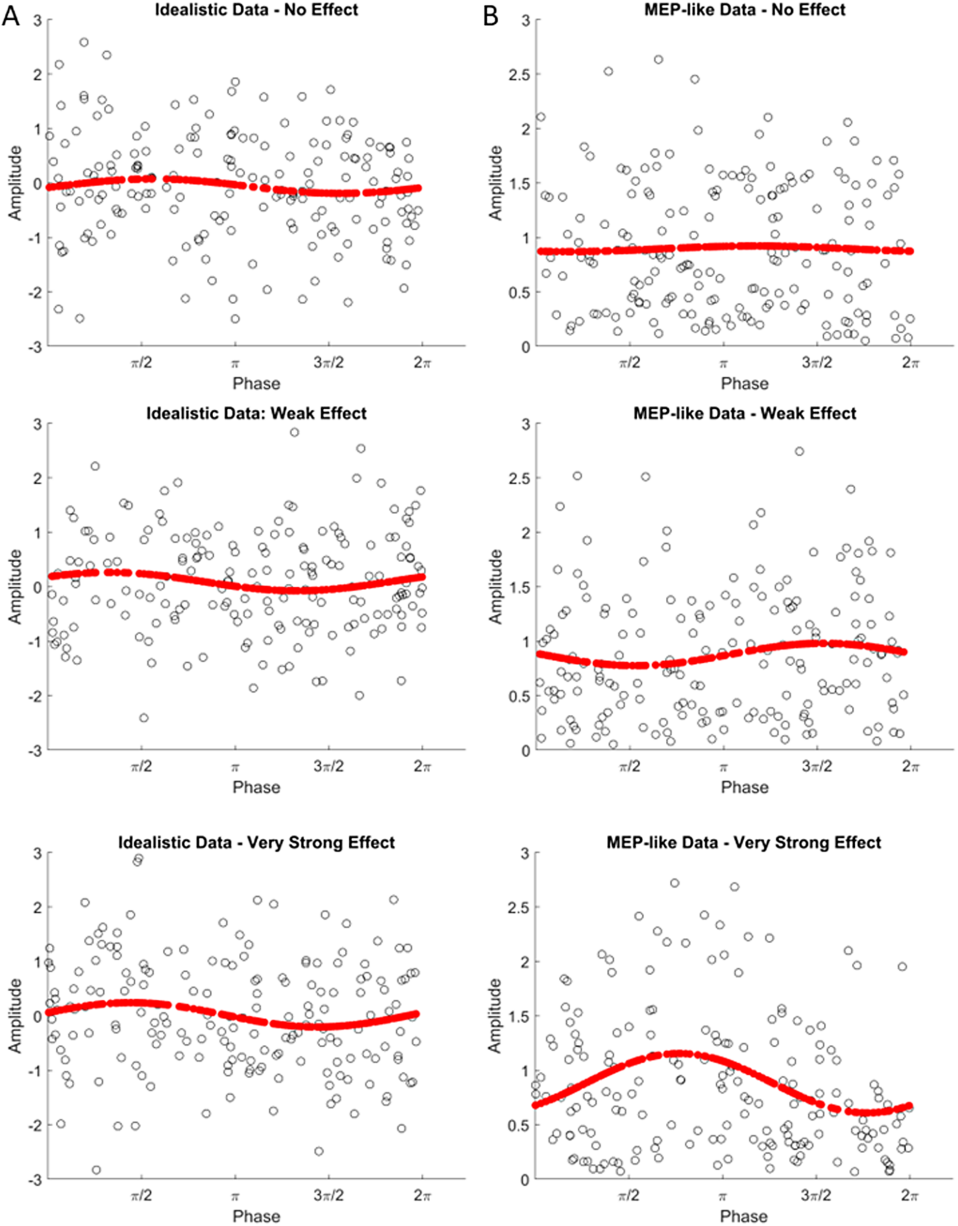
Examples of Simulated Idealistic (A) and MEP-like (B) data with FittedSinusoidal Models for the No Effect (Top), Weak Effect (Middle), andVery Strong Effect (Bottom) Scenarios. Individual data points are shownin black, whilst fitted sinusoidal models are shown in red. Theidealistic data are normally distributed around the base sine function,with the effect size scaling the sine function to peak at the specifiedamplitude. The MEP-like data are not normally distributed around thebase sine function, and so the effect size scales the variance of anormal distribution around the base sine function rather than scalingthe base sine function itself.

The effect sizes for each session were then used to generate sinusoidal data withcontinuous (i.e., randomly sampled) phase values (seeFig. 1). For the idealistic data (Fig. 1A), the effect size value scales the sine function topeak at the specified amplitude, with the data points being normally distributedaround the base sine function. For the MEP-like data (Fig. 1B), however, the effect size scales the variance of anormal distribution around the base sine function rather than scaling theamplitude of the sine function itself, since the MEP-like data explicitlyviolate the assumptions of homoscedasticity and normality. The distribution forthe MEP-like data is then folded to create a positive skew. Like MEP values,these data points, therefore, could not be negative and instead became morevariable and positive around the “peak” of tACS.

### 2.4 Analysing the simulated data

The idealistic/MEP-like data and the phase values were then pooled across theindividual sessions for each participant and a linear regression permutationanalysis was performed to calculate the individual*p*-values foreach participant (Bland & Sale,2019;Zoefel et al., 2019).The linear regression approach outlined inBlandand Sale (2019)was found to be the most sensitive when paired withpermutation analysis (Zoefel et al.,2019).Baker et al. (2021)alsofound that many paradigms where the dependent variable is derived by modelfitting show continual improvements in power as the number of trials increases,rather than reaching an asymptote where further trials provide no additionalimprovements in statistical power, as was the case for other paradigms where thedependent variable was derived by some other means. Furthermore, by modelfitting at the individual level, the individual becomes the replication unitinstead of the group (i.e., each participant can be viewed as an independentreplication of the experiment;Smith &Little, 2018).

For this linear regression analysis, an ideal (best-fitting) sinusoidal model isfirst fitted to each simulated participant’s data points based on theircorresponding phase value as described inBlandand Sale (2019). The data points are then shuffled with respect totheir phases for a total of 10000 permutations per participant and newsinusoidal models are fitted to the shuffled data. The true and shuffledsinusoidal model amplitudes are then compared, with the individual*p*-values representing the proportion of shuffled modelamplitudes that exceeded the true model amplitudes for each participant. Becausethe permutation procedure disrupts any phasic effects that may be present, theshuffled model amplitudes should be small (i.e., closer to zero) and thus, theshuffled data act as a negative control for the “true” data. Thegroup*p*-value for the experiment was then obtained by combiningthe individual*p*-values using Fisher’s method (Fisher, 1992;Zoefel et al., 2019).

### 2.5 Calculating statistical power for each scenario

This process was repeated for a total of 1000 experiments per scenario, and thestatistical power for the scenario was calculated by dividing the number ofexperiments with a significant group*p*-value (i.e.,*p*< .05) by the total number of experiments. Foreach combination of effect size and data type, the powers were averaged acrossthe different degrees of between-/within-subjects variability to form the powervalues for the primary analyses, whilst the complete power values beforeaveraging have been included asSupplementary Material.

### 2.6 Comparing statistical powers between scenarios

To determine whether the proportion of participants vs. trials significantlyinfluenced the predicted powers, chi-square goodness-of-fit tests were performedfor each combination of data type (idealistic or MEP like) and effect size (noeffect, weak, moderate, strong, or very strong).

To determine whether any combination of parameters was susceptible to over- orunder-sensitivity (i.e., if any of the powers for the no effect condition weresignificantly greater or lower than the conventional expected false discoveryrate of .05), a binomial test was performed to establish the minimum and maximumthresholds for false-positive experiments in the no effect simulations.

## 3 Results

The power values for each scenario (i.e., the proportion of significant experimentsfor each combination of effect size and number of participants/trials) aresummarised inTable 1andFigure 2. The different proportions of number ofparticipants and trials (i.e., 30 participants × 1 session, 15 participants× 2 sessions, and 10 participants × 3 sessions) were then comparedusing chi-square goodness-of-fit tests (separately for each effect size and datatype), which revealed significant improvements in statistical power as the number oftrials increases (i.e., beyond corresponding decreases in number of participants).These improvements in power were present across all effect sizes, except for thecontrol simulations with no effect, for both the idealistic (χ^2^= 0.94, 23.57, 51.76, 73.88, and 54.18 for no effect, weak, moderate, strong,and very strong, respectively;*p*= .624 for no effect and*p*< .001 for all other effect sizes) and MEP-like data(χ^2^= 0.05, 62.25, 86.53, 42.81, and 11.58,respectively;*p*= .974 for no effect,*p*< .001 for weak, moderate, and strong effects, and*p*= .003 for very strong effect).

**Table 1.** Mean estimated statistical powers for linear regression analyses usingsimulated idealistic or MEP-like sinusoidal data.

Idealistic	Effect size				
	No effect	Weak (0.075)	Moderate (0.1)	Strong (0.125)	Very strong (0.15)
30 participants × 1 session	.048	.13	.225	.358	.541
15 participants × 2 sessions	.053	.176	.338	.542	.731
10 participants × 3 sessions	.058	.221	.406	.626	.807

MEP-like	Effect size				
	No effect	Weak (0.075)	Moderate (0.1)	Strong (0.125)	Very strong (0.15)
30 participants × 1 session	.049	.196	.381	.634	.836
15 participants × 2 sessions	.051	.317	.587	.823	.948
10 participants × 3 sessions	.049	.387	.683	.881	.973

Each value represents the mean statistical power (i.e., the proportion ofsimulated experiments with a significant group*p*-value)from nine simulations with varying degrees of between- andwithin-subjects variability, each simulation comprising 1000 simulatedexperiments. Each experiment consisted of 30 sessions (60 trials each),which were divided into either 1, 2, or 3 sessions per participant, withthe trials then being pooled across sessions for each participant. Forall effect sizes (except for the control simulations with no effect),simulations with fewer participants but more sessions per participantshowed significantly greater statistical powers compared withexperiments with more participants but fewer sessions per participant.These improvements in power were present for both the idealistic(chi-square goodness-of-fit tests; χ2 = 0.94, 23.57,51.76, 73.88, and 54.18 for no effect, weak, moderate, strong, and verystrong, respectively;*p*= .624 for no effect and*p*< .001 for all other effect sizes) andMEP-like data (χ2 = 0.05, 62.25, 86.53, 42.81, and 11.58,respectively;*p*= .974 for no effect,*p*< .001 for weak, moderate, and strongeffects, and*p*= .003 for very strongeffect).

**Fig. 2.**
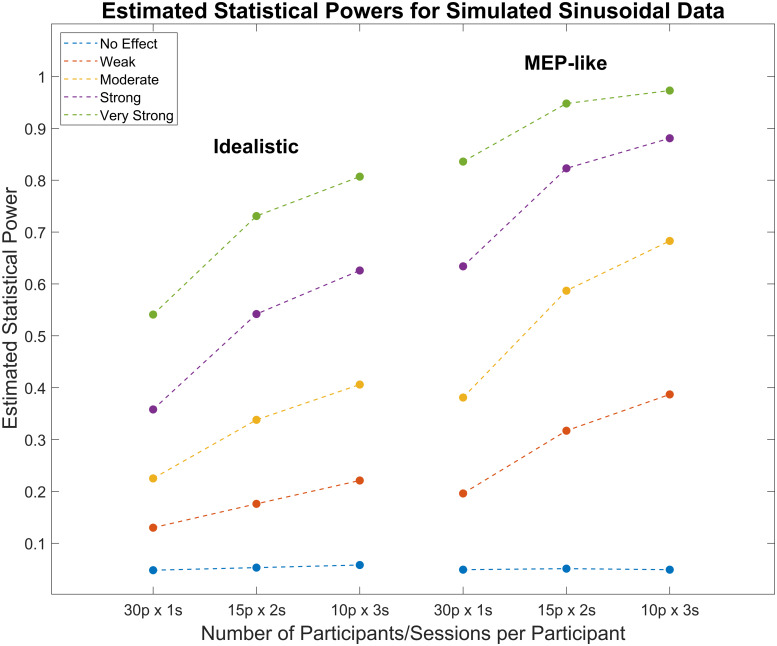
Estimated Statistical Powers for Linear Regression Analyses Using SimulatedIdealistic or MEP-like Sinusoidal Data. Simulations using idealisticsinusoidal data are shown on the left, whilst simulations using MEP-likedata are shown on the right. Each value represents the mean statisticalpower (i.e., the proportion of simulated experiments with a significantgroup*p*-value) from nine simulations with varying degreesof between- and within-subjects variability, each simulation comprising 1000simulated experiments. Each experiment consisted of 30 sessions (60 trialseach), which were divided into either 1, 2, or 3 sessions per participant,with the trials then being pooled across sessions for each participant. Eachcolour represents a different minimum effect size (blue = no effect,orange = weak, yellow = moderate, purple = strong,green = very strong). For all effect sizes (except for the controlsimulations with no effect), simulations with fewer participants but moresessions per participant showed significantly greater statistical powerscompared with experiments with more participants but fewer sessions perparticipant. These improvements in power were present for both theidealistic (chi-square goodness-of-fit tests; χ2 = 0.94,23.57, 51.76, 73.88, and 54.18 for no effect, weak, moderate, strong, andvery strong, respectively;*p*= .624 for no effectand*p*< .001 for all other effect sizes) andMEP-like data (χ2 = 0.05, 62.25, 86.53, 42.81, and 11.58,respectively;*p*= .974 for no effect,*p*< .001 for weak, moderate, and strong effects,and*p*= .003 for very strong effect).

The sensitivity of each combination of parameters to false positives was assessedusing a binomial test, which found that the minimum threshold for under-sensitivitywas 39 out of 1000 experiments (.039), with*p*= .064. Themaximum threshold for over-sensitivity was 61 out of 1000 experiments (.061), againwith*p*= .064. All of the powers from the no effectsimulations fell within these thresholds, and thus, no combination of parameters wasdeemed to be over- or under-sensitive.

## 4 Discussion

Neuroscience and, in particular, NIBS studies are often criticised for theirrelatively low statistical powers (Button et al.,2013), which have traditionally been attributed to low participant samplesizes (Lafon et al., 2017;Minarik et al., 2016;Mitra et al., 2019;Vöröslakos et al., 2018). However, it has recently beensuggested that in many cases, such as when detecting phasic effects on brainactivity, the trial sample size for each participant may be of equal, if notgreater, importance than the participant sample size itself (Baker et al., 2021;Griceet al., 2017;Normand, 2016;Rouder & Haaf, 2018;Smith & Little, 2018;Xu et al., 2018;Zoefelet al., 2019). The present stimulation study aimed to directly comparethe relative importance of participant sample size and trial sample size perparticipant for detecting phasic effects of NIBS via a linear regression permutationanalysis. To this end, we compared the statistical powers of simulated experimentswith the same number of total experiment sessions (30) but different proportions ofparticipants and number of sessions per participant (30 participants × 1session, 15 participants × 2 sessions, and 10 participants × 3sessions), with the trials being pooled across sessions for each participant. Thesesimulations were performed for two types of outcome variables (idealistic and MEPlike) and four different effect sizes (0.075—weak, 0.1—moderate,0.125—strong, 0.15—very strong), as well as a separate control with notrue effect. The chi-square goodness-of-fit tests revealed that for both data typesand all effect sizes (except for the control simulations with no effect),experiments with fewer participants but more sessions (i.e., more trials) perparticipant showed significantly greater statistical powers compared withexperiments with more participants but fewer sessions per participant, supportingour initial hypothesis. Further, the binomial test confirmed that none of thesimulated experiments were susceptible to over- or under-sensitivity.

In the case of the idealistic data, the benefit of trials over participants appearsto increase as the effect size increases. This is particularly relevant in thecontext of NIBS research, which traditionally had a history of weak effect sizesthat may still be prone to type II errors even with optimised number of trials andparticipants (Button et al., 2013;Gundlach et al., 2016;Neuling et al., 2012;Riecke, Formisano, et al., 2015;Rieckeet al., 2018;Riecke, Sack, et al.,2015;Riecke & Zoefel,2018;Wilsch et al., 2018;Zoefel et al., 2018). Despite this, our resultsclearly suggest that the benefits of trials over participants are still significanteven at weaker effect sizes. For the MEP-like data, however, it appears the benefitis of a similar magnitude irrespective of effect size. Therefore, no matter whatcapacity tACS has to modulate MEP amplitudes in a phasic manner, there is a benefitto sampling more*trials*rather than more*participants*.

There are several factors to consider when designing an experiment involving multiplesessions per participant, since it can introduce some practical issues. The first,and perhaps most impactful, of these issues is dropout/attrition: partiallycompleted datasets have to be abandoned if participants withdraw from the studybefore completing all of their sessions. It is also important to consider the lengthof time between consecutive sessions, both for the participants’ safety andto minimise any carryover effects between sessions (Alharbi et al., 2017;Brunoni &Fregni, 2011). On a similar note, if the experiment involves a task whereperformance is quantitatively assessed, it is important to consider the possibilityof training effects that may occur as the participant gains more practice with thetask with repeated sessions. Finally, researchers should strive to minimise anyother controllable variables between each participants’ sessions (time ofday, caffeine intake etc.). The severity of these challenges only worsens as thenumber of sessions per participant increases, and so researchers should considerwhat the ideal number of sessions per participant would be to minimise any practicalissues whilst still achieving the desired trial sample size. In some cases, it ispossible to achieve larger number of trials by increasing the length of eachsession, thus reducing the number of sessions needed to achieve the desired trialsample size. However, this too involves some practical issues that need to beconsidered, such as participant and/or experimenter fatigue resulting in low-qualitydata.

Despite the challenges associated with performing multiple sessions per participant,the results of these simulations suggest that the increases in statistical power areworth the small cost of these additional challenges. Furthermore, performingmultiple sessions per participant also offers some practical advantages. Forexample, the setup time at the start of each session is generally reduced after aparticipant has completed at least one session, since the participant is alreadyfamiliar with the procedure and equipment. Another advantage in subsequent sessionsthat is more specific to TMS is that it is easier to determine both the location ofthe participant’s “hot-spot” (Rossini et al., 1994) for the targeted muscle and the stimulationintensity required to consistently induce MEPs in that muscle that are around thedesired baseline amplitude (e.g., 1 mV;Cuypers etal., 2014;Ogata et al., 2019;Thies et al., 2018).

It is important to note that the simulations performed in this study assume that foreach participant, the effect sizes are approximately similar across their sessions(though this variability was manipulated across several plausible ranges forcompleteness). Further, there may also be differences in the quality of the databetween sessions, and the impact of this on statistical power is not directlyassessed in the current study. However, it is also worth noting though that whilstconsistency across sessions may be relevant for assessing the amplitude of asinusoidal effect, it is less relevant for the frequency of the effect since phaseis equivalent for any frequency. This means that individual sessions can still bepooled by phase even if there is a slight difference in the frequency of thesinusoidal effect between sessions.

## 5 Conclusions

The results of this simulation study highlight the importance of trial sample sizefor detecting phasic effects on brain activity. Our findings suggest that if thereare limitations to the number of trials that can be obtained in a single experimentsession (such as for phase-dependent TMS), conducting repeated experiment sessionson a smaller number of participants can be a useful strategy that allows researchersto obtain sufficiently large trial sample sizes and ensure accurate estimation andmodel fitting at the individual level. Although our simulation was originallydesigned in the context of our recent human tACS study (Geffen et al., 2021), effects of oscillatory phase can befound across a wide range of biomedical research fields, including but not limitedto chronobiology (Kuhlman et al., 2018),biochemistry (Uhlén & Fritz,2010), and cardiology (Tiwari et al.,2021). Further, the considerations regarding trial sample size vs.participant sample size are applicable to a wide variety of non-oscillatoryexperimental approaches in human neuroscience, psychology, and physiology. We,therefore, invite researchers to utilise the simulation code provided to aid inestimating statistical power for their own experimental designs using differentproportions of participants and trials/sessions.

## Supplementary Material

Supplementary Material

## Data Availability

The MATLAB code used to perform the simulations in this study is available athttps://osf.io/vwysk/(https://doi.org/10.17605/OSF.IO/VWYSK)
