# Supplementary material for: Balance of power: The choice between trial and participant numbers to optimise the detection of phase-dependent effects
Source: Imaging Neurosci (Camb). 2024 Nov 5;2:imag-2-00345. doi: 10.1162/imag_a_00345 (PMC12290742; doi:10.1162/imag_a_00345)
Supplement: Supplementary Material [file imag_a_00345-supp.zip › imag_a_00345-supp.pdf]

Supplementary Material

Extended Analyses for Different Levels of Between- and Within-subjects Variability

Idealistic Data

Supplementary Table 1. Estimated Statistical Powers for Linear Regression Analyses Using Simulated Idealistic Sinusoidal Data with Varying Degrees of Between- and Within-subjects Variability. *The power value for each scenario represents the proportion of experiments with a significant p-value (<0.05) from 1000 simulated experiments. Each experiment consisted of 30 sessions (60 trials each), which were divided into either 1, 2, or 3 sessions per participant, with the trials then being pooled across sessions for each participant. To simulate different degrees of between-subjects variability (BSV), the range of effect sizes for each participant was set to either ± 20%, 60%, or 100% around the mean value for low, medium, and high between-subjects variability respectively. To simulate different degrees of within-subjects variability (WSV), the effect sizes for each participant were jittered slightly between their individual sessions by either ± 10%, 20%, or 30% for low, medium, and high within-subjects variability respectively. Note that the degree of between-/within-subjects variability has no impact on the negative control simulations and so those have not been included in these extended simulations.*

Participants/Sessions	Between-/Within-subjects Variability		Effect Size			
	**BSV (1 = low, 2 = med, 3 = high)**	**WSV (1 = low, 2 = med, 3 = high)**	**Power - Weak Effect (0.075)**	**Moderate Effect (0.1)**	**Strong Effect (0.125)**	**Very Strong Effect (0.15)**
30p1s	1	1	0.116	0.169	0.287	0.446
30p1s	1	2	0.135	0.197	0.321	0.498
30p1s	1	3	0.101	0.196	0.318	0.502
30p1s	2	1	0.131	0.22	0.337	0.531
30p1s	2	2	0.119	0.231	0.343	0.524
30p1s	2	3	0.127	0.22	0.324	0.509
30p1s	3	1	0.152	0.273	0.441	0.631
30p1s	3	2	0.144	0.257	0.432	0.619
30p1s	3	3	0.147	0.262	0.416	0.61
30p1s	**Mean**		**0.130**	**0.225**	**0.358**	**0.541**
15p2s	1	1	0.162	0.287	0.467	0.686
15p2s	1	2	0.144	0.299	0.487	0.663
15p2s	1	3	0.147	0.281	0.475	0.675
15p2s	2	1	0.164	0.339	0.56	0.707
15p2s	2	2	0.187	0.321	0.496	0.705
15p2s	2	3	0.162	0.328	0.549	0.738
15p2s	3	1	0.208	0.39	0.611	0.794
15p2s	3	2	0.214	0.376	0.62	0.8
15p2s	3	3	0.198	0.419	0.612	0.815
15p2s	**Mean**		**0.176**	**0.338**	**0.542**	**0.731**

10p3s	1	1	0.203	0.374	0.571	0.785
10p3s	1	2	0.189	0.353	0.553	0.775
10p3s	1	3	0.179	0.353	0.547	0.763
10p3s	2	1	0.227	0.403	0.602	0.777
10p3s	2	2	0.228	0.385	0.637	0.805
10p3s	2	3	0.192	0.393	0.61	0.793
10p3s	3	1	0.243	0.456	0.709	0.856
10p3s	3	2	0.268	0.462	0.694	0.864
10p3s	3	3	0.262	0.475	0.707	0.843
10p3s	**Mean**		**0.221**	**0.406**	**0.626**	**0.807**

MEP-like Data

Supplementary Table 2. Estimated Statistical Powers for Linear Regression Analyses Using Simulated MEP-like Sinusoidal Data with Varying Degrees of Between- and Within-subjects Variability. *The power value for each scenario represents the proportion of experiments with a significant p-value (<0.05) from 1000 simulated experiments. Each experiment consisted of 30 sessions (60 trials each), which were divided into either 1, 2, or 3 sessions per participant, with the trials then being pooled across sessions for each participant. To simulate different degrees of between-subjects variability (BSV), the range of effect sizes for each participant was set to either ± 20%, 60%, or 100% around the mean value for low, medium, and high between-subjects variability respectively. To simulate different degrees of within-subjects variability (WSV), the effect sizes for each participant were jittered slightly between their individual sessions by either ± 10%, 20%, or 30% for low, medium, and high within-subjects variability respectively. Note that the degree of between-/within-subjects variability has no impact on the negative control simulations and so those have not been included in these extended simulations.*

**Participants/Sessions**	**Between-/Within-subjects Variability**		**Effect Size**			
	**BSV (1 = low, 2 = med, 3 = high)**	**WSV (1 = low, 2 = med, 3 = high)**	**Weak Effect (0.075)**	**Moderate Effect (0.1)**	**Strong Effect (0.125)**	**Very Strong Effect (0.15)**
30p1s	1	1	0.157	0.302	0.581	0.782
30p1s	1	2	0.183	0.34	0.547	0.788
30p1s	1	3	0.149	0.314	0.57	0.788
30p1s	2	1	0.197	0.382	0.623	0.824
30p1s	2	2	0.176	0.353	0.613	0.838
30p1s	2	3	0.204	0.383	0.62	0.828
30p1s	3	1	0.229	0.44	0.725	0.866
30p1s	3	2	0.247	0.453	0.702	0.894
30p1s	3	3	0.225	0.464	0.726	0.916
30p1s	**Mean**		**0.196**	**0.381**	**0.634**	**0.836**
15p2s	1	1	0.269	0.512	0.764	0.931
15p2s	1	2	0.278	0.502	0.77	0.934
15p2s	1	3	0.277	0.52	0.78	0.933
15p2s	2	1	0.321	0.569	0.828	0.951
15p2s	2	2	0.327	0.582	0.819	0.94

15p2s	2	3	0.285	0.601	0.822	0.955
15p2s	3	1	0.339	0.665	0.868	0.957
15p2s	3	2	0.39	0.664	0.873	0.959
15p2s	3	3	0.365	0.669	0.881	0.971
15p2s	**Mean**		**0.317**	**0.587**	**0.823**	**0.948**
10p3s	1	1	0.325	0.647	0.85	0.976
10p3s	1	2	0.362	0.625	0.86	0.98
10p3s	1	3	0.329	0.616	0.847	0.979
10p3s	2	1	0.386	0.669	0.857	0.966
10p3s	2	2	0.363	0.661	0.881	0.967
10p3s	2	3	0.375	0.657	0.884	0.97
10p3s	3	1	0.452	0.765	0.918	0.973
10p3s	3	2	0.443	0.763	0.919	0.981
10p3s	3	3	0.449	0.744	0.916	0.969
10p3s	**Mean**		**0.387**	**0.683**	**0.881**	**0.973**

Idealistic

Weak Effect

30px1s

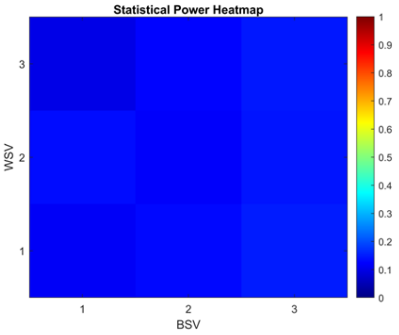

15px2s

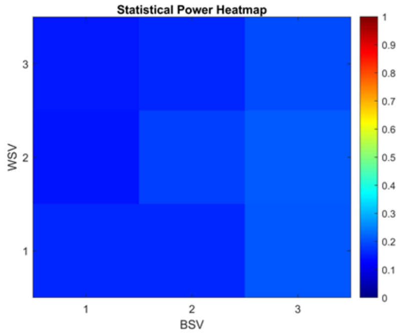

10px3s

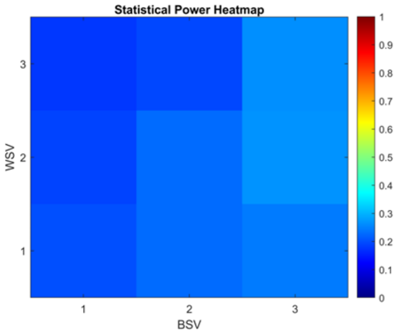

Moderate Effect

30px1s

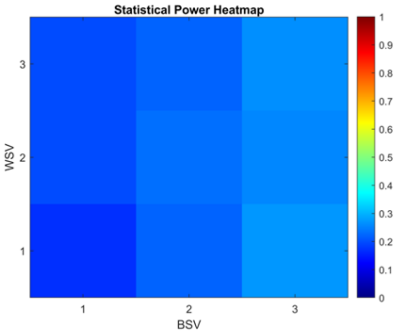

15px2s

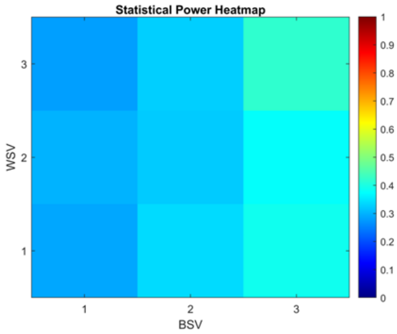

10px3s

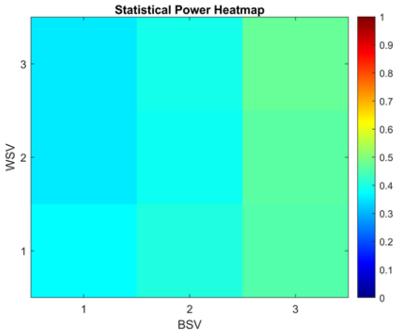

Strong Effect

30px1s

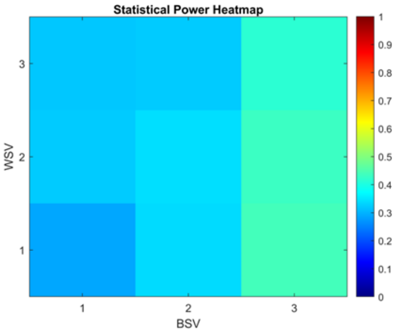

15px2s

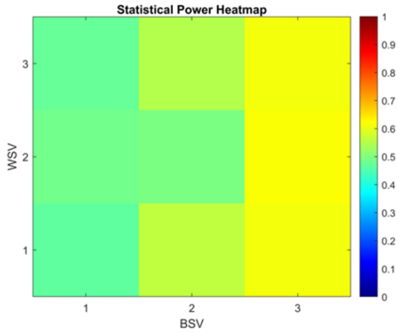

10px3s

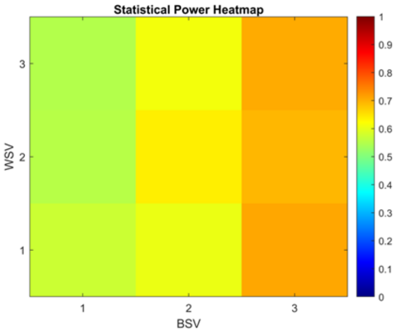

Very Strong Effect

30px1s

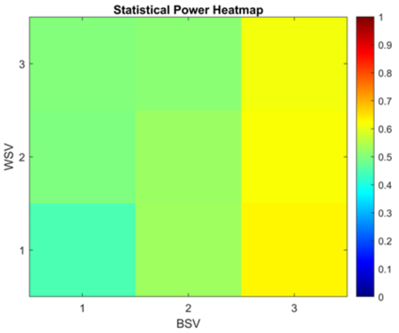

15px2s

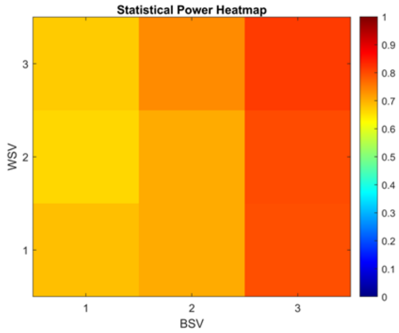

10px3s

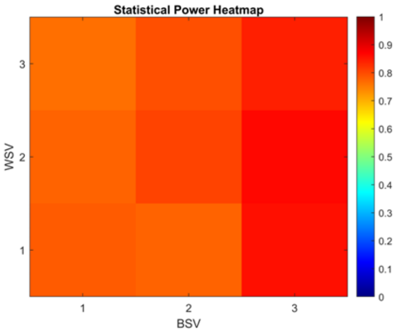

Supplementary Figure 1. Estimated Statistical Powers for Linear Regression Analyses Using Simulated Idealistic Sinusoidal Data with Varying Degrees of Between- and Within-subjects Variability. *The power value for each scenario is represented by a coloured square, with the colour indicating the estimated statistical power (i.e., the proportion of experiments with a p-value <0.05 from 1000 simulated experiments). Each experiment consisted of 30 sessions (60 trials each), which were divided into either 1, 2, or 3 sessions per participant, with the trials then being pooled across sessions for each participant. To simulate different degrees of between-subjects variability (BSV; x-axis), the range of effect sizes for each participant was set to either ± 20%, 60%, or 100% around the mean value for low, medium, and high between-subjects variability respectively. To simulate different degrees of within-subjects variability (WSV; y-axis), the effect sizes for each participant were jittered slightly between their individual sessions by either ± 10%, 20%, or 30% for low, medium, and high within-subjects variability respectively. Note that the degree of between-/within-subjects variability has no impact on the negative control simulations and so those have not been included in these extended simulations.*

MEP-like

Weak Effect

30px1s 15px2s 10px3s

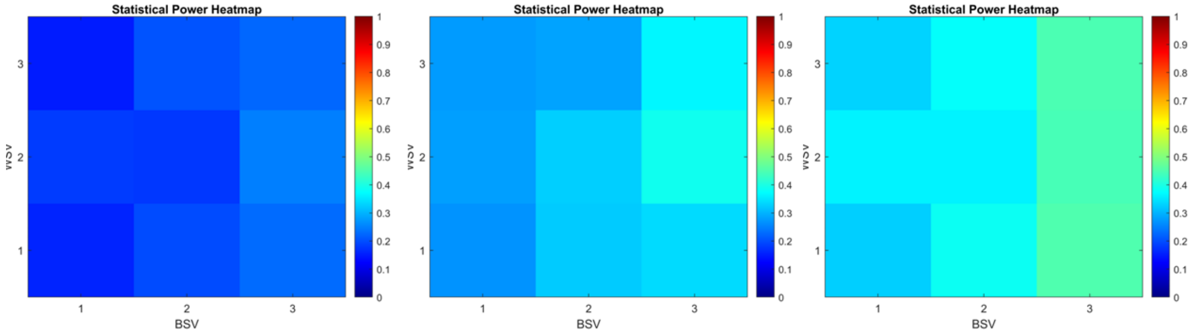

Moderate Effect

30px1s 15px2s 10px3s

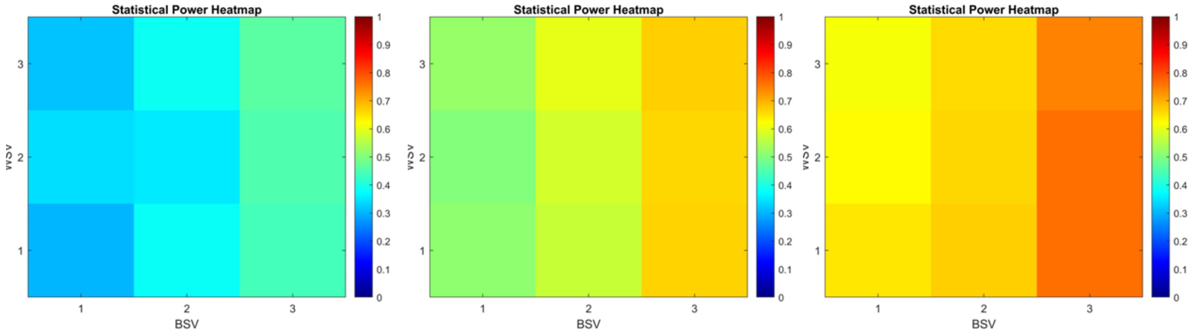

Strong Effect

30px1s 15px2s 10px3s

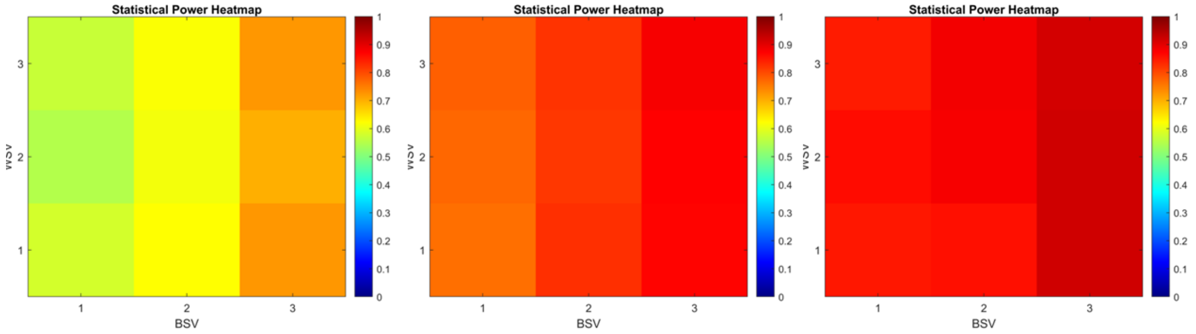

Very Strong Effect

30px1s 15px2s 10px3s

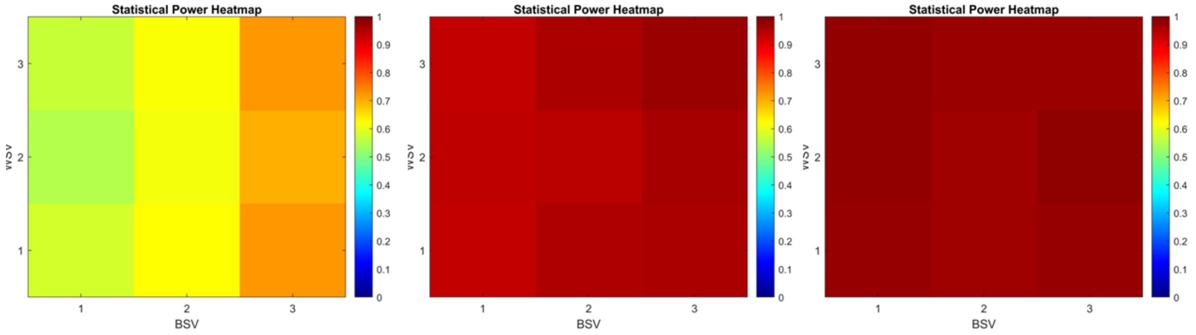

Supplementary Figure 2. Estimated Statistical Powers for Linear Regression Analyses Using Simulated MEP-like Sinusoidal Data with Varying Degrees of Between- and Within-subjects Variability. *The power value for each scenario is represented by a coloured square, with the colour indicating the estimated statistical power (i.e., the proportion of experiments with a p-value <0.05 from 1000 simulated experiments). Each experiment consisted of 30 sessions (60 trials each), which were divided into either 1, 2, or 3 sessions per participant, with the trials then being pooled across sessions for each participant. To simulate different degrees of between-subjects variability (BSV; x-axis), the range of effect sizes for each participant was set to either ± 20%, 60%, or 100% around the mean value for low, medium, and high between-subjects variability respectively. To simulate different degrees of within-subjects variability (WSV; y-axis), the effect sizes for each participant were jittered slightly between their individual sessions by either ± 10%, 20%, or 30% for low, medium, and high within-subjects variability respectively. Note that the degree of between-/within-subjects variability has no impact on the negative control simulations and so those have not been included in these extended simulations.*
